# Genetic suppressor of *fd-gogat1* reveals crosstalk among brassinosteroids, photorespiration, and amino acid metabolism

**DOI:** 10.3389/fpls.2025.1680431

**Published:** 2025-12-01

**Authors:** Katherine A. Klimpel, Annika Findlay, Sannidhi Menon, Leah Cytron-Walker, Caylynn A. Dedow, Lazlo Camou, Michela Cadarso, Meghan F. McGuire, Jeff Pietroske, Daniel Idowu, Michael Busche, Jacob O. Brunkard

**Affiliations:** Laboratory of Genetics, University of Wisconsin – Madison, Madison, WI, United States

**Keywords:** glutamate synthase, photorespiration, gibberellin, brassinosteroid, plant hormones, plant metabolism, plant genetics

## Abstract

**Introduction:**

A classical forward genetic screen for Arabidopsis mutants with altered morphology identified a pleiotropic mutant, *orbiculata (orb1)*, that has phenotypes including rounded leaves, chlorosis, and reduced growth. *orb1* mapped to one of the Arabidopsis genes that encodes glutamate synthase, *fd-gogat1* (ferredoxin-dependent glutamine oxoglutarate aminotransferase or Fd-GOGAT).

**Methods:**

To discover why this glutamate synthase impacts development, we conducted a forward genetic screen for suppressors of *orb1*. In the primary mutagenized generation, we identified a dominant mutant, which we call *Lettuce*, that rescues *orb1* chlorosis but causes new pleiotropic defects that closely resemble the classical Arabidopsis *cabbage* and *dwarf* mutants that are defective in brassinosteroid or gibberellin signaling. Here, we take a chemical genetic approach to phenocopy *Lettuce* and investigate how gibberellins and brassinosteroids impact the development and physiology of *fd-gogat1*.

**Results:**

We found that inhibiting brassinosteroid synthesis significantly increases chlorophyll content in *fd-gogat1*, which is chlorotic due to defects in the photorespiratory pathway.

**Discussion:**

This discovery highlights how crosstalk among phytohormones (brassinosteroids) and core metabolic processes (amino acid biosynthesis and photorespiration) converge to regulate plant development and physiology.

## 1 Introduction

Plants are photoautotrophs that can synthesize all twenty proteinogenic amino acids from inorganic precursors, primarily CO_2_ assimilated by RuBisCO, NH_4_^+^ assimilated by glutamine synthetase, and SO_4_^2-^ assimilated by O-acetylserine thiollyase. The enzymes responsible for amino acid metabolism in plants have been elucidated over several decades using a combination of biochemical and genetic approaches. Amino acid synthesis in plants is deeply intertwined with other metabolic pathways, however, which complicates genetic analysis due to pleiotropic effects of disrupting the genes that encode enzymes involved in amino acid metabolism. Moreover, many enzymes are encoded by several paralogues in plant genomes that may be semi-redundant or may play specialized, subfunctionalized roles in metabolism (Maeda, 2019).

**Figure 1:**
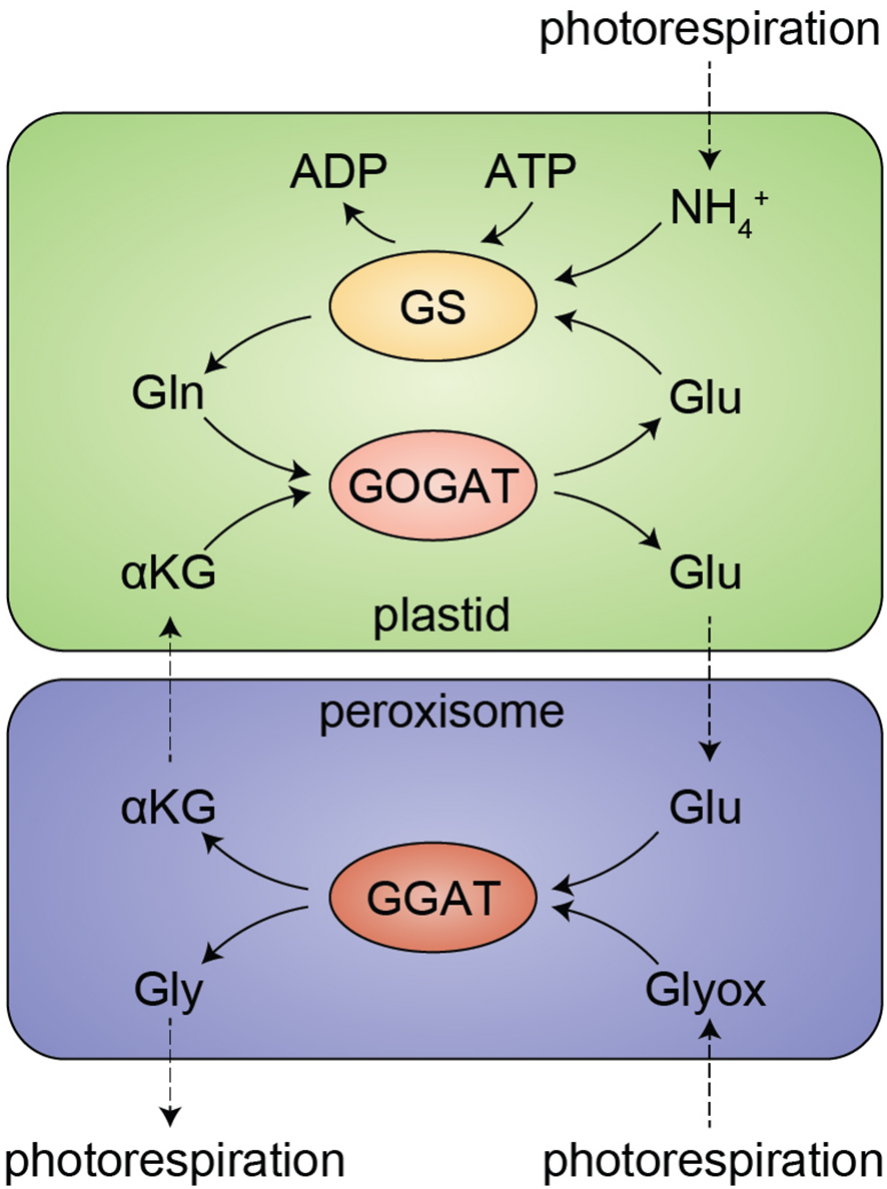
The GS/GOGAT cycle and photorespiration in leaves. Ammonium (NH_4_^+^), a byproduct of photorespiration, is salvaged by glutamine synthase (GS) in an ATP-dependent reaction to synthesize glutamine (Gln) from glutamate (Glu). Gln is then condensed with α-ketoglutarate (αKG, 2-oxoglutarate, or 2-OG) by glutamine oxoglutarate aminotransferase (GOGAT), yielding two Glu. In *fd-gogat1* mutants lacking this recycling pathway, Glu levels in leaves are depleted within minutes during the day (Somerville and Ogren, 1980), leading to toxic overaccumulation of NH_4_^+^. Moreover, as part of the salvage pathway that rescues carbon during photorespiration, Glu is directly consumed in the peroxisome, where glutamate:glyoxylate aminotransferase (GGAT) converts Glu and glyoxylate (Glyox) to αKG and glycine (Gly). Thus, the GS/GOGAT cycle is required to rescue both nitrogen and carbon under photorespiratory conditions.

Illustrating this complexity, in the model plant *Arabidopsis thaliana*, there are three *bona fide* glutamate synthases each encoded by their own genes: FERREDOXIN-DEPENDENT GLUTAMATE SYNTHASE 1 (Fd-GOGAT1), Fd-GOGAT2, and NICOTINAMIDE ADENINE DINUCLEOTIDE-DEPENDENT GLUTAMATE SYNTHASE 1 (NADH-GOGAT1). There are an additional three genes that encode semi-redundant GLUTAMATE DEHYDROGENASE (GDH) enzymes, which are biochemically capable of synthesizing glutamate from α-ketoglutarate (αKG) and ammonium *in vitro*. *In vivo*, however, GDHs are understood to primarily catalyze the reverse reaction, deaminating glutamate to αKG to support the tricarboxylic acid cycle, and GDHs are therefore not typically involved in glutamate synthesis (Fontaine et al., 2012). GOGATs work intimately with glutamine synthetase (GS) to assimilate nitrogen in the GS-GOGAT cycle: GOGAT makes glutamate, which GS condenses with ammonia to yield glutamine (**Figure 1**). *Fd-GOGAT2* and *NADH-GOGAT1* are highly expressed in roots, where they drive nitrogen assimilation from the soil in the GS/GOGAT cycle (Lancien et al., 2002). *Fd-GOGAT1* is instead highly expressed in leaves, where it plays a critical role in reassimilating carbon and nitrogen that are lost during photorespiration (Coschigano et al., 1998).

Photorespiration occurs in normal air conditions when RuBisCO oxygenates ribulose bisphosphate, yielding the waste product 2-phosphoglycolate (2-PG), instead of carboxylating ribulose bisphosphate, yielding the sugar precursor 3-phosphoglycerate (3-PG). The carbons “wasted” to 2-PG are recovered through photorespiration, a complicated metabolic process that requires glutamate, alanine, various other metabolites, and over a dozen enzymes that are spread across the cell in the cytosol, plastids, perxoisomes, and mitochondria (Bauwe et al., 2010; Peterhansel et al., 2010; Eisenhut et al., 2019; Timm and Hagemann, 2020). Photorespiration yields 3-PG and also releases NH_3_, which is then re-assimilated by the GS-GOGAT cycle. *fd-gogat1* mutants accumulate toxic levels of NH_3_ in leaves and are unable to sustain photosynthesis, resulting in smaller, yellow plants when grown in standard conditions.

Arabidopsis first came to prominence among modern molecular biologists through the forward genetic screens that established the mechanisms of photorespiratory metabolism (Somerville and Ogren, 1980, p. 198; Somerville, 2001), but Arabidopsis already had a long history of investigation by developmental geneticists who researched how genes can influence leaf shape (Micol, 2009). Although leaf shape mutant phenotypes are often caused by disruptions in regulatory genes that encode, e.g., transcription factors that drive patterning or phytohormone signaling pathways (Moon and Hake, 2011), a surprising number of classical leaf shape mutants mapped to genes involved in primary metabolism or ribosome biogenesis (Martinez et al., 2025). For example, *orbiculata 1 (orb1)*, a mutant with dramatically rounder leaves than wild-type siblings, is caused by loss of *fd-gogat1* (Muñoz-Nortes et al., 2017).

Here, we set out to investigate how disrupting *FD-GOGAT1* causes the *orb1* leaf shape phenotype by conducting a forward genetic screen for *orb1* suppressors. Unexpectedly, we discovered a strong, dominant, and ultimately lethal suppressor of the *fd-gogat1* chlorotic phenotype in the mutagen-treated M1 population. Based on phenotypic comparison of this suppressor to well-studied Arabidopsis mutants, we explored how two major phytohormones, gibberellins (gibberellic acids, GAs) and brassinosteroids (BRs), impact the development and physiology of *fd-gogat1* mutants. We show that inhibiting BR biosynthesis partially restores chlorophyll levels in *fd-gogat1* plants, demonstrating how amino acid metabolism in plants intersects with other metabolic networks (such as photorespiration) and phytohormone signaling to determine physiological and developmental outcomes.

## 2 Materials and methods

### 2.1 Plant materials and growth conditions

Unless otherwise stated, plants were grown under standard conditions with 16 h day/8 h night at ~120 µE/m^2^s, 23°C, and 50% humidity. The *fd-gogat1* line, SALK_011035C (previously called *orb1-4* (Muñoz-Nortes et al., 2017)), and the Col-0 (wild-type) line were obtained from the Arabidopsis Biological Resource Center.

### 2.2 Forward genetic *fd-gogat1* suppressor screen

**Figure 2:**
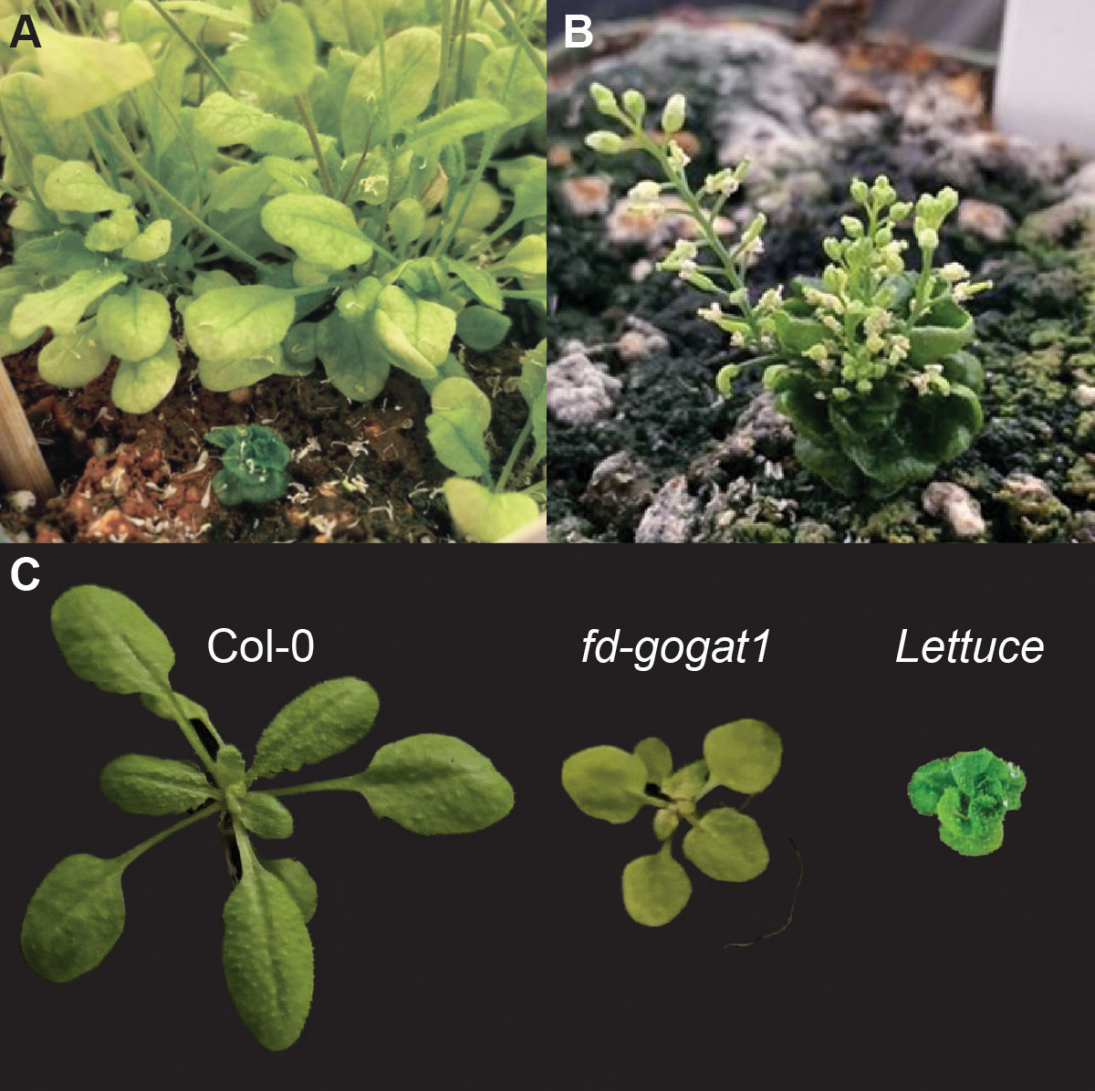
*Lettuce* suppresses *fd-gogat1*. **(A)***Lettuce* was discovered in the EMS-mutagenized M1 generation during a screen for genetic suppressors of *fd-gogat1*. *Lettuce* is shown here alongside its *fd-gogat1* siblings in the M1 generation. **(B)***Lettuce* continued to grow and eventually flowered weeks after *fd-gogat1* siblings had completed their life cycle. *Lettuce* flowers were stunted, did not produce any pollen, and could not be fertilized by Col-0 or *fd-gogat1* pollen. **(C)** Comparison of Col-0, *fd-gogat1*, and the double mutant *Lettuce;fd-gogat1* rosettes.

Mutagenesis of *fd-gogat1* seeds was carried out as previously described (Gillmor and Lukowitz, 2020). 100 mg *fd-gogat1* seeds (~5,000 seeds) were weighed and then washed with 0.01% Tween 20 (VWR 97063-872) in Milli-Q H_2_O for 15 minutes. Tween 20 solution was removed and seeds were washed with Milli-Q H_2_O four times until no more bubbles formed. 40 mL of Milli-Q H_2_O were added to the seeds, which were then placed at 4°C on a rocker for gentle agitation overnight. The next day, Milli-Q H_2_O was removed from seeds. 1.2% Ethyl methanesulfonate (EMS) (Sigma-Aldrich M0880-5G) solution was prepared and poured into pre-treated seeds. Seeds were next placed on a rocker at room temperature to incubate for three hours under gentle agitation, ensuring full coverage of seeds by EMS solution. Seeds were washed 3 times in the fume hood with Milli-Q H_2_O and an additional 10 washes with tap water were performed outside of the hood over the span of 1 hour. Seeds were then suspended in 40 mL of a dilute agar slurry (0.4% and 0.1% agar solutions mixed in a 50/50 ratio) and left at room temperature overnight. 1 mL of seed slurry was drizzled on top of wet potted soil to get about 100 seeds planted per pot. Seeds were grown in a growth chamber under long day 16-hour light/8-hour dark conditions with a light intensity of 120 µE/m^2^s, at 23°C and 50% humidity. Plants were observed and screened over time for phenotypes to indicate EMS was successful (**Figures 2**).

### 2.3 Phytohormone and chemical inhibitor treatments

Seeds from *Arabidopsis thaliana* mutant *fd-gogat1* (obtained from the Arabidopsis Biological Resource Center, line SALK_011035C) and Col-0 were sterilized using 30% bleach and 0.1% tween for 15 minutes and washed with sterile Milli-Q H_2_O 5 times to remove any excess bleach solution. Seeds were stratified at 4°C in 1 mL sterile Milli-Q H_2_O for 48 hours. Seeds were plated on ½ MS (RPI M10200-50.0) with 1% sucrose (RPI S24065-5000.0) and 0.8% agar (Fisher BioReagents BP9744-5). In addition to mock-treated “control” plates, seeds were sown on plates with gibberellic acid 3 (GA) (Dot Scientific DSG32020-5) or paclobutrazol (PBZ) (TCI America P2299) at 10 µM and 30 µM concentrations, propiconazole (PCZ) (Cayman Chemical 18853) at 1 µM and 5 µM concentrations, brassinazole (BRZ) (TCI America B2829) at 1 µM, 5 µM, or 10 µM concentrations, or brassinolide (BL) (Cayman Chemical 21594) at 0.5 µM or 1 µM concentrations. Plates were sealed with micropore tape and placed in growth chambers under a long day 16-hour light/8-hour dark cycle with a light intensity of 150-180 µmol/m^2^s for 14 or 20 days.

### 2.4 Chlorophyll quantifications

Seedlings were pooled, weighed (~100 mg of tissue per pool), and placed into tubes with 3 steel beads, 8 plants per pool for mock-treated and GA plates or 16 plants per pool for BL, BRZ, and PCZ plates, and flash frozen in liquid nitrogen. Plant tissue was ground using a homogenizer at 1,500 rpm for 1 minute. Chlorophyll was extracted by washing ground tissue with 100% acetone, vortexing for 30 seconds, centrifuging at 10,000 x *g* for 1 min, and then removing and saving the supernatant. This process was repeated 5 times to extract all of the chlorophyll from each sample. 50 µL extract was diluted in 800 µL ice cold 80% acetone solution and centrifuged for 5 min at 14,000 x *g*. UV-vis spectrometry was conducted on each sample using wavelengths of 647 nm, 664 nm, and 750 nm.

### 2.5 Growth measurements and data analysis

**Figure 3:**
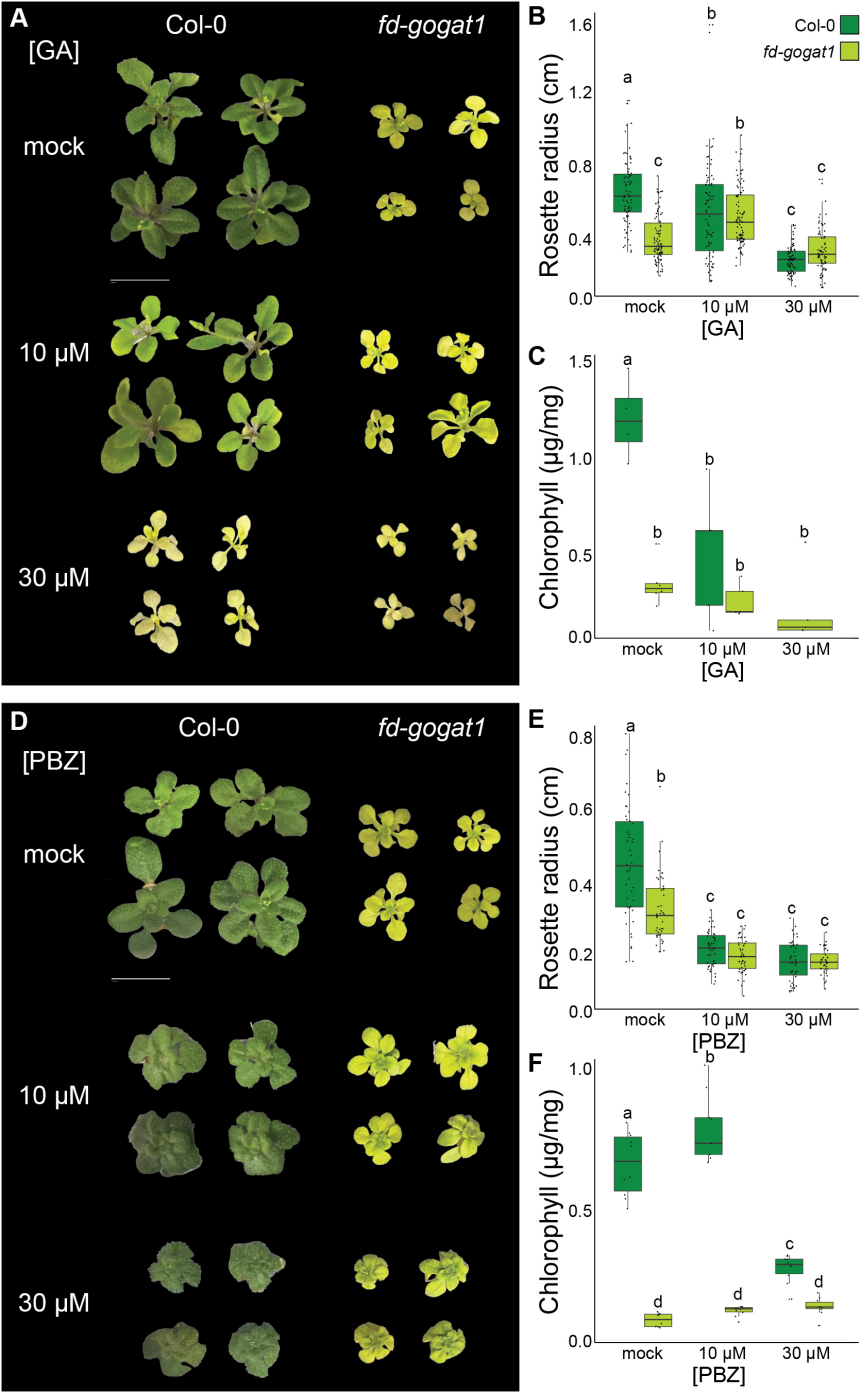
Inhibiting GA synthesis only moderately rescues *fd-gogat1* chlorosis. **(A)** Representative images of Col-0 and *fd-gogat1* seedlings grown on plates with indicated GA concentrations. **(B)** GA somewhat reduced Col-0 rosette diameter and had minimal effect on *fd-gogat1* rosette diameter. **(C)** GA significantly reduced chlorophyll levels in both Col-0 or *fd-gogat1*. **(D)** Representative images of Col-0 and *fd-gogat1* seedlings grown on plates with indicated PBZ concentrations. **(E)** PBZ significantly reduced rosette diameter in both genotypes, with a more pronounced effect on Col-0. **(F)** 30 µM PBZ decreased chlorophyll in Col-0 and significantly increased chlorophyll levels in *fd-gogat1*, although the effect on *fd-gogat1* was very slight. Letters indicate significance groups as determined by Tukey’s HSD test, *p* < 0.05.

**Figure 4:**
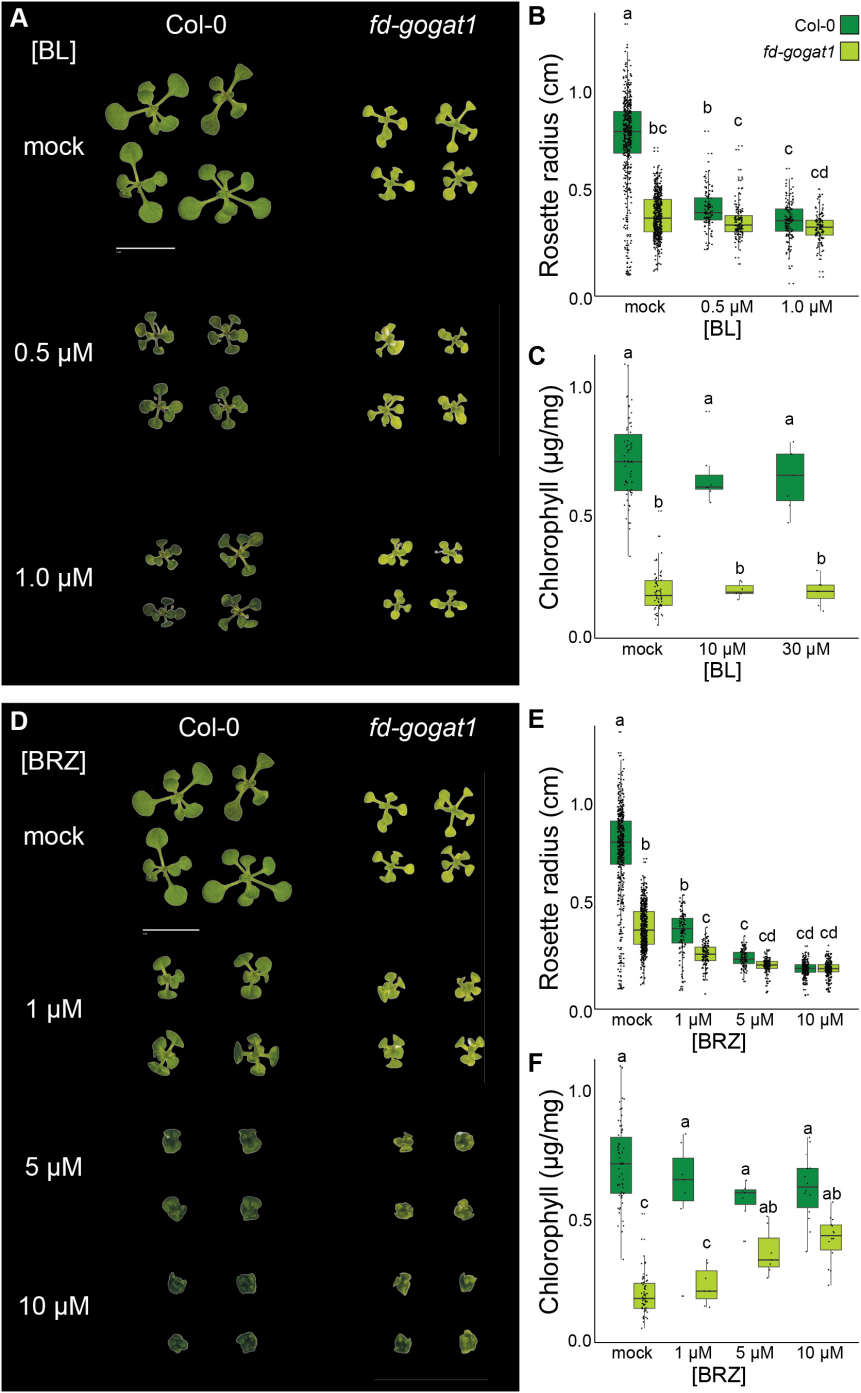
Inhibiting BR synthesis phenocopies *Lettuce* and partially rescues *fd-gogat1* chlorosis. **(A)** Representative images of Col-0 and *fd-gogat1* seedlings grown on plates with indicated BL concentrations. **(B)** BL significantly reduced Col-0 rosette diameter but had no substantial effect on *fd-gogat1*. **(C)** BL had not substantial effect on chlorophyll levels in Col-0 or *fd-gogat1*. **(D)** Representative images of Col-0 and *fd-gogat1* seedlings grown on plates with indicated BRZ concentrations. *fd-gogat1* mutants treated with 5 µM to 10 µM BRZ closely resembled the *Lettuce* suppressor mutant. **(E)** BRZ significantly reduced rosette diameter in both genotypes, with a more pronounced effect on Col-0. **(F)** BRZ slightly decreased chlorophyll in Col-0 but significantly increased chlorophyll levels in *fd-gogat1*. Letters indicate significance groups as determined by Tukey’s HSD test, *p* < 0.05.

Plates were photographed beside a ruler for scaling under laboratory light on bench top. Rosette radius was then measured in ImageJ. Leaf number was counted at the indicated time, excluding cotyledons (which developed prior to treatments). Total chlorophyll concentrations and chlorophyll a/b ratios were calculated using the standard formula (Porra et al., 1989). Data were analyzed with R software using Student’s *t*-test for chlorophyll comparisons or ANOVA with Fisher’s exact test for rosette radius. Statistically-significant groupings indicated in **Figures 3** and **4** were determined using Tukey’s HSD test with confidence level 0.95. Plant figures were made by removing the background and replacing with a black background. The brightness and contrast of the plants were uniformly adjusted across all images, with no other modifications. Scales were set based on a ruler in each picture.

## 3 Results

### 3.1 *Lettuce* suppresses *fd-gogat1* chlorosis

To identify genetic mechanisms that drive the pleiotropic *orbiculata* syndrome in *fd-gogat1* mutants, we conducted a forward genetic suppressor screen. *fd-gogat1* seeds were mutagenized with ethyl methanesulfonate (EMS), which causes G/C to A/T transitions (Greene et al., 2003). The point mutations introduced by EMS can have diverse effects on gene function, including (but not limited to) missense mutations, nonsense mutations, or mutation of regulatory features (like splice sites) that broadly disrupt gene function. To validate that the EMS mutagenesis was successful, we screened the M1 generation for mosaic leaf color phenotypes; to ensure an efficient Arabidopsis genetic screen, we expect to observe as many as ~1% of plants with mutant sectors that have yellow or white leaves (Maple and Møller, 2007). During this analysis, we made a surprising discovery: a single plant with dramatically altered phenotypes from its siblings, which we named *Lettuce* (**Figure 2A**).

*Lettuce* was small, compact, and bright green, with curling leaves, slowed shoot development, multiple inflorescence stems, and delayed flowering compared to its *fd-gogat1* siblings (**Figure 2B**). None of the bright green leaves of *Lettuce* yellowed or senesced after flowering, unlike Col-0 and *fd-gogat1* plants. We confirmed that *Lettuce* was homozygous for the SALK_011035 T-DNA insertion in *fd-gogat1* and was therefore a true suppressor and not a contaminant. *Lettuce* was sterile: flowers produced no pollen and could not be fertilized with pollen from other plants, which prevented us from propagating the genotype for deeper phenotypic analysis and genetic mapping. Since *Lettuce* was discovered in the M1 generation, it is almost certainly caused by a dominant mutation, which are less frequently encountered in genetic screens but can be powerful tools for discovery, especially when a dominant allele is also epistatic to redundant paralogues.

Since we could not map *Lettuce* to a causal mutation, we instead probed the literature for similar mutant phenotypes that could illuminate how *Lettuce* suppresses the chlorotic phenotype in *fd-gogat1*. Although there are many possible candidates, we noted that *Lettuce* is remarkably similar to mutants defective in GA and BR signaling. For example, Arabidopsis *gid1a;gid1b;gid1c* mutants lacking all three paralogues of *GIBBERELLIN INSENSITIVE DWARF 1 (GID1)*, which encode the GA receptors (Murase et al., 2008), are extremely small, slow-growing, dark green, and infertile (Griffiths et al., 2006). Comparable phenotypes are also observed in the Arabidopsis *ga20ox1;ga20ox2;ga20ox3* (Plackett et al., 2012) mutants that lack most of the GIBBERELLIN 20-OXIDASES that are required for synthesis of bioactive GA. *Lettuce* is also strikingly reminiscent of the *cabbage (cbb)* mutants (Kauschmann et al., 1996), which were shown to encode the BR receptor (*cbb2* is an allele of BRASSINOSTEROID INSENSITIVE 1; other alleles are called *bri1*, *bin1*, and *dwarf2*) (Clouse et al., 1996; Li and Chory, 1997; Choe et al., 1998; Wang et al., 2001; Nam and Li, 2002; Hothorn et al., 2011; She et al., 2011) and enzymes involved in BR synthesis (*cbb1*, also called *dwarf1* and *diminuto*, encodes a sterol C-24 reductase; *cbb3*, also called *dwarf3* and *constitutive photomorphogenic dwarf*, encodes a cytochrome P450 enzyme, CYP90A) (Szekeres et al., 1996; Klahre et al., 1998; Choe et al., 1999a; Ohnishi et al., 2012). *Lettuce* similarly resembled other mutants defective in BR signaling, including the semidominant mutant *bin2 (brassinosteroid insensitive 2*, also called *dwarf12*) that constitutively represses BR responses (Li et al., 2001; Choe et al., 2002), and other mutants defective in BR synthesis, such as *dwarf4* (which encodes CYP90B1) (Choe et al., 1998; Fujita et al., 2006), *dwarf5* (Choe et al., 2000), *det2* (*de-etiolated 2*, also called *dwarf6)* (Chory et al., 1991; Noguchi et al., 1999), and *dwarf7* (also called *sterol1* and *boule1*) (Gachotte et al., 1996; Choe et al., 1999b; Catterou et al., 2001). Inspired by the close similarities between these mutants and *Lettuce*, we speculated that disrupting GA and/or BR synthesis might be sufficient to phenocopy *Lettuce* and increase chlorophyll levels in *fd-gogat1* mutants.

### 3.2 Inhibiting GA biosynthesis slightly rescues chlorophyll levels in *fd-gogat1*

Building on our observation that the *Lettuce* suppressor of *fd-gogat1* resembles mutants defective in GA signaling and biosynthesis, we tested how treating plants with GA or paclobutrazol (PBZ), a selective inhibitor of GA biosynthesis (Hedden and Graebe, 1985), impacts *fd-gogat1* growth, development, and physiology. We grew *fd-gogat1* and Col-0 plants on 0.8% agar plates with ½ × Murashige & Skoog (MS) nutrients and 1% sucrose, plus GA, PBZ, or mock treatment. 20 days after germination, seedlings were photographed to measure rosette diameter and seedlings of each genotype from different plates were pooled for chlorophyll extraction and quantification (**Figure 3**). GA and PBZ were both supplied at either 10 µM or 30 µM, which are common effective concentrations for these hormones; in trial experiments, we tested lower concentrations and observed no substantial effects.

As expected, *fd-gogat1* mutants were significantly smaller (*p* < 10^-15^, *n* ≥ 77) with significantly less chlorophyll (*p* < 0.01, *n* ≥ 4) than Col-0 plants (**Figure 3**). When treated with 10 µM GA, however, the rosette diameter was indistinguishable between the two genotypes (*p* = 0.50, *n* ≥ 78), because 10 µM GA increased *fd-gogat1* rosette diameter but had the opposite effect on Col-0 plants (**Figure 3B**). 30 µM GA slightly reduced the rosette diameter of both genotypes compared to treatment with 10 µM GA, but *fd-gogat1* mutants were significantly larger with this treatment than wild-type plants (*p* < 0.01, *n* ≥ 77) (**Figure 3B**). The GA biosynthesis inhibitor, PBZ, decreased rosette diameter in both genotypes (*p* < 10^-11^, *n* ≥ 40), with a marginally stronger effect at 30 µM (**Figure 3E**).

Whereas GA had opposite effects on rosette diameter for the two genotypes, GA reduced chlorophyll levels in both *fd-gogat1* and Col-0 (**Figure 3C**). In contrast, PBZ slightly but significantly increased chlorophyll levels in *fd-gogat1* (*p* < 0.01, *n* ≥ 5 pools of seedlings) (**Figure 3F**), supporting the hypothesis that disrupting GA biosynthesis could partially suppress *fd-gogat1* chlorosis. PBZ also increased chlorophyll levels in Col-0 plants when treated with the lower concentration of 10 µM PBZ (*p* = 0.04, *n* ≥ 4), but treatment with 30 µM PBZ drastically reduced Col-0 chlorophyll levels (*p* < 10^-6^, *n* ≥ 4) (**Figure 3F**).

Overall, these experiments validated that a chemical genetic approach can replicate mutant analysis, since PBZ-treated Col-0 plants closely resembled the phenotypes of previously-studied GA signaling mutants, such as *gid1a;gid1b;gid1c* and *ga20ox1;ga20ox2;ga20ox3*. PBZ-treated *fd-gogat1* mutants, however, did not closely resemble the *Lettuce* suppressor, suggesting that a disruption to GA biosynthesis or signaling is unlikely to be the cause of *Lettuce* phenotypes. PBZ did increase chlorophyll in *fd-gogat1* mutants, hinting at possible crosstalk between GA biosynthesis and photorespiration, but the effect was very minor compared to the bright green phenotype of *Lettuce*.

### 3.3 Inhibiting brassinosteroid biosynthesis phenocopies the *lettuce* suppressor of *fd-gogat1*

Next, based on the similarity of *Lettuce* phenotypes to the *cabbage* and *dwarf* BR biosynthesis mutants, we tested how treating plants with the biologically-active BR brassinolide (BL) or the highly selective BR biosynthesis inhibitor brassinazole (BRZ) (Asami et al., 2000) impacted *fd-gogat1* phenotypes compared to Col-0 and mock-treated controls. Again, we grew *fd-gogat1* and Col-0 plants on 0.8% agar plates with ½ × MS nutrients and 1% sucrose, plus BL, BRZ, or mock treatment. 13 days after germination, seedlings were photographed to measure rosette diameter and then ~15 seedlings from each plate were pooled for chlorophyll extraction and quantification (**Figure 4**). BL was supplied at 0.5 µM or 1.0 µM and BRZ was supplied at 1.0 µM, 5.0 µM, or 10 µM, which are commonly used dose ranges for these chemicals.

Under these experimental conditions, BL significantly reduced rosette diameter for both genotypes (*p* < 0.01, *n* ≥ 115), although the effect was more pronounced for Col-0 than for *fd-gogat1* mutants (**Figure 4B**). BL had no significant effect on chlorophyll accumulation in either genotype, however (*p* > 0.20, *n* ≥ 7 pools of seedlings) (**Figure 4C**). Inhibiting BR biosynthesis with the selective inhibitor BRZ caused similar phenotypes in both Col-0 and *fd-gogat1* that very closely resembled the *Lettuce* mutant (**Figure 4D**). BRZ significantly reduced rosette diameter in both genotypes (*p* < 10^-10^, *n* ≥ 110) (**Figure 4E**). The higher concentrations of BRZ (5 µM and 10 µM) also mildly reduced chlorophyll levels in Col-0 plants (*p* < 0.05, *n* ≥ 7 pools of seedlings) (**Figure 4F**). Oppositely, these concentrations of BRZ dramatically increased chlorophyll levels in *fd-gogat1* (*p* < 0.01, *n* ≥ 7 pools of seedlings), an effect that was strikingly similar to the *Lettuce* mutant (**Figure 4F**).

To validate and confirm these findings, we conducted an additional experiment with propiconazole (PCZ), a broad cytochrome P450 inhibitor that is thought to primarily interfere with BR biosynthesis in plants (Hartwig et al., 2012). Col-0 and *fd-gogat1 s*eedlings were grown for 20 days on 0.8% agar plates with ½ × MS nutrients and 1% sucrose, supplemented with either 1.0 µM PCZ, 5.0 µM PCZ, or mock controls. 5.0 µM PCZ was sufficient to significantly increase chlorophyll levels 1.8-fold in *fd-gogat1* (*p* = 0.02, *n* ≥ 5 pools of seedlings), with the opposite effect on Col-0 plants, significantly decreasing chlorophyll levels 2.0-fold (*p* < 0.01, *n* ≥ 5 pools of seedlings). This supported the hypothesis that inhibiting BR biosynthesis partially rescues chlorophyll accumulation in the *fd-gogat1* photorespiration mutant.

## 4 Discussion

Photorespiration is responsible for major metabolic inefficiencies in plants, cutting net photosynthetic efficiency by ~50% (Zhu et al., 2008) and reducing many crop yields by ~20-40% (Walker et al., 2016). To overcome these losses, some photosynthetic lineages evolved carbon-concentrating mechanisms that reduce photorespiration by isolating RuBisCO in high CO_2/_low O_2_ environments, such as C4 photosynthesis found in several plant lineages, including the major crops maize, sorghum, and sugarcane (Sage, 2004; Kellogg, 2013; Schlüter and Weber, 2020); CAM photosynthesis, also found in several plant lineages, including many agave, pineapple, and cacti (Bräutigam et al., 2017); and pyrenoids in algae (He et al., 2023). Restricting or bypassing photorespiration, inspired by these evolutionary examples, is a promising target for breeders and synthetic biologists seeking to establish the resilient, high-yielding crops we will need for a sustainable agricultural future (Walker et al., 2016; Springmann et al., 2018; Bailey-Serres et al., 2019; Eisenhut et al., 2019; South et al., 2019; Lutt and Brunkard, 2022; Meacham-Hensold et al., 2024; Hadjikakou et al., 2025).

As demonstrated with the *fd-gogat1* mutant, however, photorespiration is deeply intertwined with manifold core metabolic and developmental pathways, including amino acid biosynthesis, phytohormone signaling, redox homeostasis, and even leaf patterning. A deeper understanding of the crosstalk among these interconnected processes will be needed to guide efforts to engineer photorespiration bypasses in crops. Here, we showed that inhibiting brassinosteroid synthesis can rescue the chlorotic *fd-gogat1* phenotype at the cost of reducing plant size and fertility. This trade-off could be mitigated by modulating BR signaling in specific cell types or in response to specific cues, rather than broadly inhibiting BR biosynthesis. For instance, ubiquitously overexpressing the BR response transcription factor, *BRASSINAZOLE RESISTANT 1 (BZR1)*, drastically reduces fertility and photosynthetic efficiency, but overexpressing *BZR1* exclusively in bundle sheath cells increases chloroplast area without negative trade-offs (Cackett et al., 2025).

Forward genetic screens in Arabidopsis for mutants that are visibly unhealthy in low CO_2_ environments but healthy in high CO_2_ environments (Somerville and Ogren, 1980) were among the first demonstrations that Arabidopsis genetics could be leveraged to resolve fundamental questions about plant physiology and biochemistry (Somerville, 2001), paving the way for the burst in Arabidopsis research in the 1990s. Despite >40 years of extensive research on these mutants, however, we still do not have a unified mechanistic understanding of why mutants defective in photorespiration exhibit such diverse phenotypes, ranging from mild growth defects to complete lethality (Timm and Bauwe, 2013). These phenotypes are not only suppressed by growing plants in high CO_2_ environments or, as we have shown here for the chlorotic phenotype of *fd-gogat1*, by inhibiting brassinosteroid synthesis, but sometimes by other conditions, including fluctuating light environments (von Bismarck et al., 2023). Whereas the suppression of photorespiration by high CO_2_ environments is easily explained, the suppressive effects of other treatments is less obvious; inhibiting BR synthesis, for example, might be directly regulating expression of chlorophyll biosynthesis and photosynthesis-associated genes or indirectly impacting *fd-gogat1* photorespiration phenotypes through more complex effects on, e.g., nitrogen uptake, recycling, and metabolism, which are known to be sensitive to BR (Wang et al., 2019; Xing et al., 2022; Yadav et al., 2023; Yang et al., 2024). A combination of genetic and physiological approaches to unravel the functional roles of photorespiration in metabolism (Timm et al., 2024) and the signaling pathways that contribute to photorespiratory mutant phenotypes will be needed to build strong predictive models of how changing environmental CO_2_/O_2_ levels will impact plant health and agricultural yields.

*fd-gogat1* is not only defective in photorespiration, but also in amino acid metabolism due to its role in glutamate synthesis from glutamine and 2-oxoglutarate. Across all eukaryotes, amino acid metabolism is monitored by the TARGET OF RAPAMYCIN (TOR), a serine/threonine kinase that is activated when conditions are favorable (Valvezan and Manning, 2019; Brunkard, 2020). TOR activity coordinates metabolism with nutrient availability by, among other mechanisms, driving ribosome biogenesis and protein synthesis when amino acids, nucleotides, and ATP are abundant in cells (Xiong et al., 2013; Scarpin et al., 2020, 2022; Busche et al., 2021). In mammals, TOR is particularly sensitive to the levels of essential amino acids that heterotrophs rely on consuming in their diets, especially leucine, arginine, and methionine (Jewell and Guan, 2013; Lutt and Brunkard, 2022). Several molecular sensors and mediating signal transduction pathways upstream of TOR have been identified in mammals and yeast (Goul et al., 2023), but these sensors and mediators are not conserved to plants (Brunkard, 2020). A handful of studies have demonstrated that TOR does sense amino acids in plants (Cao et al., 2019; Liu et al., 2021), but the molecular mechanisms have not yet been defined. Here, we illustrated a major challenge for biologists seeking to understand how plants sense and respond to amino acids: mutating one of the genes responsible for glutamate synthesis causes broad, unintended side effects. Adding to this complexity, the plant TOR network has evolved to interact various plant-specific signaling networks, including phytohormones like BR (Zhang et al., 2016; Liao et al., 2023) and metabolite transport via plasmodesmata (Brunkard et al., 2020). Establishing growth conditions, genetic approaches, or other methods to disentangle amino acid synthesis from other metabolic pathways and signaling networks will be needed to eventually elucidate how TOR monitors amino acid levels in plants.

## Data Availability

The datasets presented in this study can be found in online repositories. The names of the repository/repositories and accession number(s) can be found in the article/supplementary material.
